# Effects of Chinese herbal medicine on plasma glucose, protein and energy metabolism in sheep

**DOI:** 10.1186/2049-1891-4-51

**Published:** 2013-12-18

**Authors:** Xi Liang, Kyota Yamazaki, Mohammad Kamruzzaman, Xue Bi, Arvinda Panthee, Hiroaki Sano

**Affiliations:** 1 Faculty of Agriculture, Iwate University, 3-18-8 Ueda, Morioka 020-8550, Iwate, Japan

**Keywords:** Chinese herbal medicine, Glucose metabolism, Heat production, Protein metabolism, Ruminal fermentation characteristics, Sheep

## Abstract

**Background:**

The use of antibiotics in animal diets is facing negative feedback due to the hidden danger of drug residues to human health. Traditional Chinese herbal medicine has been used to replace antibiotics in the past two decades and played an increasingly important role in livestock production. The present study was carried out to assess the feeding effects of a traditional nourishing Chinese herbal medicine mixture on kinetics of plasma glucose, protein and energy metabolism in sheep. Ruminal fermentation characteristics were also determined.

**Methods:**

Four sheep were fed on either mixed hay (MH-diet) or MH-diet supplemented with 2% of Chinese herbal medicine (mixture of Astragalus root, Angelica root and Atractylodes rhizome; CHM-diet) over two 35-day periods using a crossover design. The turnover rate of plasma glucose was measured with an isotope dilution method using [U-^13^C]glucose. The rates of plasma leucine turnover and leucine oxidation, whole body protein synthesis (WBPS) and metabolic heat production were measured using the [1-^13^C]leucine dilution and open circuit calorimetry.

**Results:**

Body weight gain of sheep was higher (*P = 0.03*) for CHM-diet than for MH-diet. Rumen pH was lower (*P = 0.02*), concentration of rumen total volatile fatty acid tended to be higher (*P = 0.05*) and acetate was higher (*P = 0.04*) for CHM-diet than for MH-diet. Turnover rates of plasma glucose and leucine did not differ between diets. Oxidation rate of leucine tended to be higher (*P = 0.06*) for CHM-diet than for MH-diet, but the WBPS did not differ between diets. Metabolic heat production tended to be greater (*P = 0.05*) for CHM-diet than for MH-diet.

**Conclusions:**

The sheep fed on CHM-diet had a higher body weight gain and showed positive impacts on rumen fermentation and energy metabolism without resulting in any adverse response. Therefore, these results suggested that the Chinese herbal medicine mixture should be considered as a potential feed additive for sheep.

## Background

Antibiotics have been widely used as feed additive in livestock production for more than 50 yr [1]. They have played a very important role in helping animals to prevent diseases and enhance productivity. However, their use in animal diets has also brought along the hidden danger of drug residues to human health [2]. At present, the use of antibiotic growth promoters in animal industry is restricted in the European Union. It is likely that the restrictions on the use of antibiotics in animal husbandry will spread to the rest of the world due to the increasing public concern on human health. Therefore, replacing antibiotics with alternative feed additives has become the specific research interest to animal scientists.

Chinese herbs as the traditional medicine have been widely used in East Asian countries. For thousands of years, they have made great contributions to the maintenance of human health. Most of the Chinese herbal medicine comes from the different parts of perennial herbs, such as the leaves, roots and stems. It has been well-known that Chinese herbal medicine contains the bioactive components which have anti-bacterial activity, anti-inflammatory properties and immune enhancing effects [3]. Because of the natural origin, Chinese herbal medicine will not cause excessive drug residue or toxicity and thus it can be considered as a safe and suitable substitute for antibiotics in animal feeding. In recent years, a lot of Chinese herbal medicine has already been reported to promote growth and boost immune system in pigs, chickens and other animals [4-6].

It was considered that such numerous Chinese herbal medicine provide a great potential for practical application and some of them could be used as alternative feed additive for ruminants. Until now, little information is available regarding the performance of Chinese herbal medicine on nutrients and energy metabolism in ruminants. We hypothesized that Chinese herbal medicine might be beneficial to nutrients and energy metabolism in ruminants due to its bioactive properties. Therefore, the present study was carried out to assess the effects of feeding a traditional nourishing Chinese herbal medicine mixture on kinetics of plasma glucose, protein and energy metabolism in sheep.

## Methods

### Animals, diets and management

The handling of animals, including cannulation and blood collection, was reviewed and approved by the Animal Care Committee of Iwate University. All experimental procedures were performed without any noticeable stress to the animals.

According to the classical Chinese pharmacopoeia, a traditional nourishing Chinese herbal medicine mixture was utilized in this study. Three herbs were mixed in proportion as 55% of Astragalus root (*Astragalus membranaceus*), 27% of Angelica root (*Angelica sinensis*) and 18% of Atractylodes rhizome (*Atractylodes lancea*). The herbs are known to be rich in polysaccharides (Astragalus root) and essential oils (Angelica root and Atractylodes rhizome). In humans, the Chinese herbal medicine mixture is commonly used as a health regulator to remove tiredness and comfort stress by inducing hematopoiesis.

Four crossbred (Corriedale × Suffolk) shorn wethers, aged 7 mo on average, weighing 29 ± 2 kg, were used in the experiment. The sheep were assigned to two dietary treatments, including either mixed hay (MH-diet) of orchardgrass (*Dactylis glomerata*) and reed canarygrass (*Phalaris arundinacea*) offered at maintenance level [7] or MH-diet supplemented with 2% of Chinese herbal medicine mixture (CHM-diet). The chemical compositions of mixed hay and Chinese herbal medicine on air dry matter basis are shown in Table 1. The sheep received mixed hay at 67 g/kgBW^0.75^/d for both dietary treatments. The experiment was performed using a crossover design over two 35-day periods. The sheep were housed in individual pens in an animal room during the first four weeks of the experiment for adaptation. Then animals were moved to metabolic cages in a controlled environment house at an air temperature of 23 ± 1°C, with lighting present from 08:00 h to 22:00 h. Two sheep were fed on the MH-diet during the first period and then fed on the CHM-diet during the second period. The other two sheep were subjected to the dietary treatments in the reverse order. The animals were given either diet twice a day at 08:30 h and 20:30 h, and they commonly consumed all the diets which were fed within 1 h. Water was available *ad libitum*. The sheep were weighted at the start of experiment, on day 15 and 29, and at the end of each dietary treatment.

**Table 1:** Chemical composition of experimental diets

Items*	**MH**	**CHM**
Dry matter, %	88.3	91.2
CP, %	11.9	10.8
NDF, %	65.1	47.2
ME, kcal/g	1.79	N/A

*Values are presented on air dry matter basis.

MH = mixed hay of orchardgrass and reed canarygrass; CHM = Chinese herbal medicine mixture; CP = crude protein; NDF = neutral detergent fiber; ME = metabolizable energy; N/A = not available.

### Experimental procedures

On day 33 of each dietary treatment, rumen fluid (30 ml) was collected through a stomach tube before feeding, 3 and 6 h after feeding. After determination of pH values of rumen fluid with a pH meter (HM-20E, Toa Electronics Ltd., Japan), the rumen fluid was centrifuged at 8,000 × *g* for 10 min at 4°C (RS-18IV, Tomy, Japan). Then 1 ml of supernatant was mixed with 1 ml of 0.1 N HCl. The prepared samples and the residuals of rumen fluid were stored at −30°C for the determinations of rumen ammonia (NH_3_) and volatile fatty acid (VFA), respectively.

Two sheep were subjected to the experimental procedures to determine plasma glucose, protein and energy metabolism on day 34 and 35, respectively. The other two sheep were determined in the reverse order. The turnover rate of plasma glucose was measured with an isotope dilution method using [U-^13^C]glucose. The rates of plasma leucine turnover and leucine oxidation, whole body protein synthesis and metabolic heat production were measured using the [1-^13^C]leucine dilution and open circuit calorimetry. Two catheters, one for isotope infusion and the other for blood collection were inserted into the left and right jugular veins on the morning of day 34 of each dietary treatment. The catheters were filled with sterile solution of 3.8% trisodium citrate.

At 12:00 h on the day of [U-^13^C]glucose dilution, 3.0 μmol/kgBW^0.75^ of [U-^13^C]glucose (D-glucose-^13^C, 99 atom% excess ^13^C; Cambridge Isotope Laboratories, USA) dissolved in saline solution (0.9% sodium chloride) was injected into the jugular catheter for infusion as a priming dose. After the priming injection, [U-^13^C]glucose was then continuously infused by multichannel peristaltic pumps (AC-2120, Atto, Japan) at rate of 3.0 μmol/kg^0.75^/h through the same catheters for 4 h. Blood samples were taken from the sampling catheter immediately before (12 ml) and at 30-min intervals (6 ml) during the last 120 min of isotope infusion. The collected samples were immediately transferred into centrifuge tubes containing heparin sodium and were stored in crushed ice. Plasma was separated from blood samples by means of centrifugation at 10,000 × *g* for 10 min at 4°C and then stored at −30°C for further analysis.

At 11:30 h on the day of [1-^13^C]leucine dilution, the sheep were fitted with a clear head chamber (approximately 0.2 m^3^) to collect the samples of exhaled gas. After collecting gaseous samples in the pre-infusion period, 7.2 μmol/kgBW^0.75^ of [1-^13^C]leucine (L-leucine-1-^13^C, 99 atom% excess ^13^C; Cambridge Isotope Laboratories, USA) and 3.5 μmol/kgBW^0.75^ of NaH^13^CO_3_ (sodium bicarbonate-^13^C, 99.2 atom% excess ^13^C; Cambridge Isotope Laboratories, USA) dissolved in saline solution were injected into the infusion catheter as a priming dose. Then, [1-^13^C]leucine was continuously infused by multichannel peristaltic pumps at rates of 7.2 μmol/kg^0.75^/h through the same catheters for 6 h. Blood samples were taken from the sampling catheter immediately before (12 ml) and at 30-min intervals (6 ml) during the last 120 min of isotope infusion. The catheters were removed at the end of isotope infusions on day 35 of each dietary treatment.

Oxygen (O_2_) consumption and carbon dioxide (CO_2_) production were monitored continuously for 30 min at the end of [1-^13^C]leucine infusion using a metabolic monitor (Coast Electronics, UK). An aliquot of exhaled CO_2_ was collected in 4 ml of 1 N NaOH for 30 min immediately before and three times during the last 90 min of [1-^13^C]leucine infusion to measure the enrichment of exhaled CO_2_ derived from leucine oxidation. The CO_2_ samples were stored at −30°C for further analysis.

### Chemical analysis

Analysis of chemical components of the diets was performed according to Association of Official Analytical Chemists [8]. Nitrogen contents in diets were analyzed using the Foss Kjeltec System (Kjeltec 2300, Foss, Sweden). The NDF contents in diets were measured according to Van Soest *et al*. [9] using the Foss Analytical FiberCap™ system (Foss, Sweden).

Concentrations of rumen NH_3_ were determined with a colorimetric method [10] using a spectrophotometer (V-630, JASCO, Japan). Concentrations of rumen VFA were measured using a gas chromatography (HP-5890, Hewlett Packard, USA) after steam distillation.

Concentrations of plasma free amino acids and urea were determined using an automatic amino acid analyzer (JLC-500/V, JEOL, Japan). Concentrations of plasma non-esterified fatty acid (NEFA) were enzymatically determined using a diagnostic kit (NEFA C, Wako Pure Chemicals, Japan).

Plasma glucose was derivatized to glucose aldonitrile pentaacetate according to the procedure of Tesrng and Kalhan [11] with slight modifications by Fujita *et al*. [12]. Enrichments of plasma [U-^13^C]glucose were measured by gas chromatography mass spectrometry (QP-2010, Shimadzu, Japan). Concentrations of plasma glucose were enzymatically determined with a glucose oxidase method of Huggett and Nixon [13].

Concentrations of plasma α-ketoisocaproic acid (α-KIC) and enrichments of plasma α-[1-^13^C]KIC were measured by gas chromatography mass spectrometry according to the procedures of Rocchiccioli *et al*. [14] and Calder and Smith [15]. The exhaled CO_2_ captured in 1 N NaOH solutions was released by adding 6 N H_2_SO_4_ into the samples in rubber capped vials under a vacuum condition, and the isotopic abundance of exhaled ^13^CO_2_ was determined using a gas chromatography-combustion-isotope ratio mass spectrometric system (DELTA^plus^, Thermo Electron, USA).

### Calculations

The turnover rates of plasma glucose and leucine (GluTR and LeuTR, respectively) as well as leucine oxidation rate (LeuOX) were calculated using the equations given by Wolfe [16]:

$$\mathrm{TR}=I\times \left(1/E‒1\right)$$

Where, *I* represents the infusion rates of [U-^13^C]glucose and [1-^13^C]leucine, and *E* represents the plasma isotopic enrichments of [U-^13^C]glucose and α-[1-^13^C]KIC during the steady state, respectively. In the present study, the enrichment of plasma α-[1-^13^C]KIC was used to calculate LeuTR instead of plasma [1-^13^C]leucine, because it is a more useful indicator to determine leucine metabolism [17].

$$\text{LeuOX}=E_{\text{CO}2}/E_{\mathrm{KIC}}/0.81\times V_{\text{CO}2}$$

Where, *Eco*_*2*_ is the isotopic enrichment of exhaled ^13^CO_2_ and *Vco*_*2*_ is the CO_2_ production rate. The recovery fraction of exhaled CO_2_ production in the animal body was estimated to be 0.81 [18]. Whole body protein synthesis (WBPS) was calculated using the following equation as described by Wolfe *et al*. [19]:

$$\begin{array}{ll} \text{WBPS} & =\left(\text{LeuTR}-\text{LeuOX}\right)\\ & \;/\text{leucine}\;\text{concentration}\;\text{in}\;\text{carcass}\;\text{protein} \end{array}$$

Leucine concentration in carcass protein (66 g/kg) was used as described by Harris *et al*. [20].

Heat production (HP) was calculated based on the Brouwer’s equation [21] using O_2_ consumption and CO_2_ production as described by Young *et al*. [22]:

$$\text{HP}=3.866\times V_{O2}+1.2\times V_{\mathrm{CO}2}$$

Where, *Vo*_*2*_ is the O_2_ consumption rate and *Vco*_*2*_ is the CO_2_ production rate.

### Statistical analysis

Statistical analysis was conducted according to the MIXED procedure of SAS [23]. The analysis of variance was used to test the effects of period and diet, and sheep were the random effect. Results were considered significant at *P* < *0.05* level, and tendency was at *0.05* ≤ *P* < *0.10*.

## Results

Mean values with standard error of the mean (SEM) were given. Body weight gain of sheep and the ruminal fermentation parameters are shown in Table 2. The sheep fed on CHM-diet had a greater (*P = 0.03*) daily weight gain than those fed on MH-diet. Rumen pH was lower (*P = 0.02*) for CHM-diet than for MH-diet. Concentration of rumen NH_3_ remained similar between diets. Concentration of rumen total VFA tended to be higher (*P = 0.05*) and acetate was higher (*P = 0.04*) for CHM-diet than for MH-diet.

**Table 2:** Effects of Chinese herbal medicine on body weight gain and ruminal fermentation characteristics in sheep

**Items**	**MH-diet**	**CHM-diet**	**SEM**	***P*-value**
BW gain, g/day	16	25	6	0.03
pH	6.99	6.84	0.04	0.02
NH_3_, mmol/L	10.7	11.2	0.6	0.65
Total VFA, mmol/L	86.2	92.8	3.2	0.05
Acetate, mmol/L	60.5	64.5	2.1	0.04
Propionate, mmol/L	17.2	19.1	0.9	0.35
Iso-butyrate, mmol/L	0.6	0.7	0.01	0.15
Butyrate, mmol/L	6.5	7.2	0.3	0.34
Iso-valerate, mmol/L	0.8	0.9	0.02	0.21
Valerate, mmol/L	0.5	0.5	0.01	0.12

MH-diet = mixed hay of orchardgrass and reed canarygrass; CHM-diet = MH-diet supplemented with 2% of Chinese herbal medicine mixture; SEM = standard error of the mean; BW = body weight; VFA = volatile fatty acids.

Plasma free amino acids, urea and NEFA determined in the pre-infusion period of isotope dilution method are presented in Table 3. Concentration of plasma aspartic acid was higher (*P = 0.02*) and those of threonine, leucine, phenylalanine, alanine, proline and total amino acid tended to be higher (*P* < *0.10*) for CHM-diet than for MH-diet. Concentrations of plasma urea and NEFA did not differ between diets.

**Table 3:** Effects of Chinese herbal medicine on concentrations of plasma metabolites at pre-infusion period in sheep

**Items**	**MH-diet**	**CHM-diet**	**SEM**	***P*-value**
Essential amino acids, μmol/L				
Threonine	258	282	20	0.07
Valine	268	315	17	0.13
Methionine	25	27	2	0.52
Iso-leucine	89	109	7	0.21
Leucine	129	156	9	0.09
Phenylalanine	61	68	4	0.06
Histidine	58	64	3	0.44
Lysine	112	130	9	0.16
Non essential amino acids, μmol/L				
Aspartic acid	7.2	8.1	1.1	0.02
Serine	141	159	6	0.30
Asparagine	52	60	3	0.24
Glutamic acid	78	90	4	0.15
Glutamine	311	349	17	0.32
Glycine	467	501	25	0.39
Alanine	190	207	9	0.08
Tyrosine	90	95	7	0.37
Tryptophan	32	47	4	0.34
Arginine	141	149	11	0.19
Proline	107	124	8	0.09
Total amino acid	2616	2940	268	0.07
Urea, mmol/L	6.5	7.2	0.8	0.10
NEFA, mEq/L	0.16	0.14	0.03	0.12

MH-diet = mixed hay of orchardgrass and reed canarygrass; CHM-diet = MH-diet supplemented with 2% of Chinese herbal medicine mixture; SEM = standard error of the mean; NEFA = non-esterified fatty acid.

**Figure 1:**
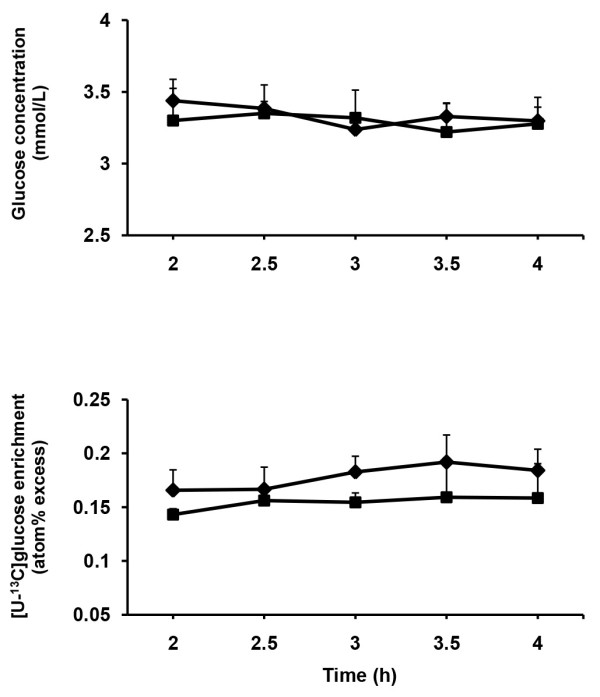
**Time course changes of plasma glucose concentration and [U-**^**13**^**C]glucose enrichment.** Time course of changes in plasma glucose concentration and [U-^13^C]glucose enrichment during the last 2 h of [U-^13^C]glucose infusion in sheep fed two different diets (◆ = MH-diet, ■ = CHM-diet).

Concentration of plasma glucose and enrichment of plasma [U-^13^C]glucose remained constant during the last 2 h of isotope infusion for each dietary treatment (Figure 1). Concentration of plasma glucose and GluTR did not differ between diets (Table 4).

**Table 4:** Effects of Chinese herbal medicine on plasma glucose, protein and energy metabolism in sheep

**Items**	**MH-diet**	**CHM-diet**	**SEM**	***P*-value**
Glucose concentration, mmol/L	3.36	3.33	0.15	0.84
GluTR, mmol/kg^0.75^/h	1.45	1.56	0.04	0.17
α-KIC concentration, μmol/L	9.5	8.9	1.3	0.42
LeuTR, μmol/kg^0.75^/h	398	422	23	0.16
LeuOX, μmol/kg^0.75^/h	102	160	11	0.06
WBPS, g/kgBW^0.75^/d	14.1	12.5	1.4	0.15
HP, kcal/kg^0.75^/h	3.94	4.25	0.07	0.05

MH-diet = mixed hay of orchardgrass and reed canarygrass; CHM-diet = MH-diet supplemented with 2% of Chinese herbal medicine mixture; SEM = standard error of the mean; GluTR = turnover rate of plasma glucose; α-KIC = α-ketoisocaproic acid; LeuTR = turnover rate of plasma leucine; LeuOX = oxidation rate of leucine; WBPS = whole body protein synthesis; HP = heat production.

**Figure 2:**
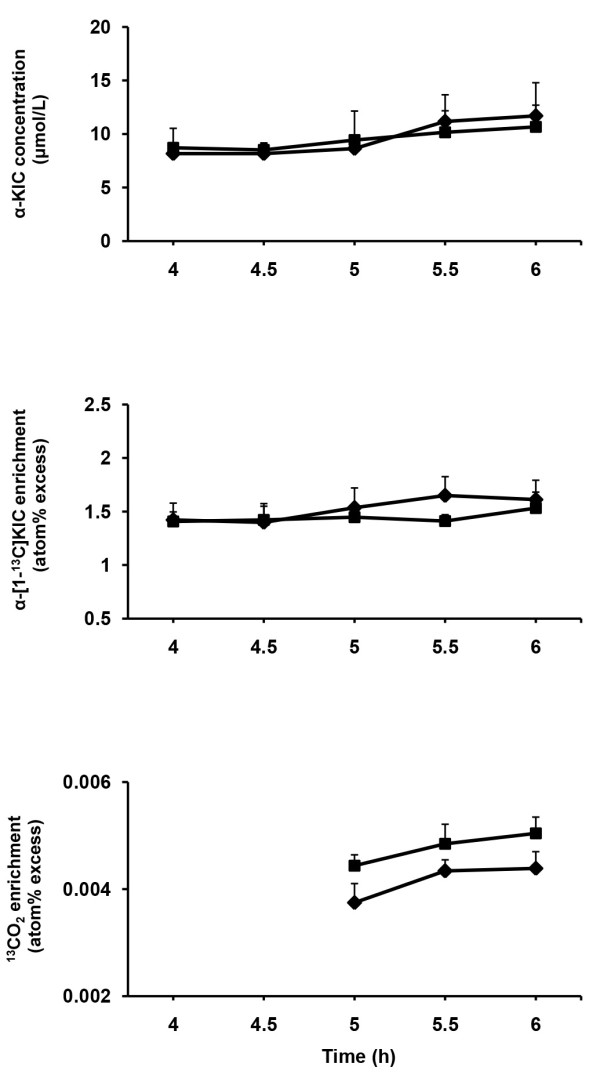
**Time course changes of plasma α-KIC concentration and enrichments of plasma α-[1-**^**13**^**C]KIC and exhaled **^**13**^**CO**_**2**_**.** Time course of changes in plasma α-ketoisocaproic acid (α-KIC) concentration and enrichments of plasma α-[1-^13^C]KIC and exhaled ^13^CO_2_ during the latter period of [1-^13^C]leucine infusion in sheep fed two different diets (◆ = MH-diet, ■ = CHM-diet).

Concentration of plasma α-KIC and enrichments of plasma α-[1-^13^C]KIC and exhaled ^13^CO_2_ were stable during the latter period of [1-^13^C]leucine infusion (Figure 2). Concentration of plasma α-KIC remained similar for both dietary treatments. Plasma LeuTR did not differ between diets. The LeuOX tended to be greater (*P = 0.06*) for CHM-diet than for MH-diet, but the WBPS did not differ between diets. Metabolic heat production tended to be greater (*P = 0.05*) for CHM-diet than for MH-diet (Table 4).

## Discussion

### Ruminal fermentation characteristics

In ruminants, the rumen is a large fermentation chamber which plays a very important role in feed digestion. The VFA is produced as the digestive product of dietary carbohydrates through microbial fermentation in the rumen. In the present study, concentration of rumen total VFA tended to be higher and acetate was higher in sheep fed on CHM-diet than on MH-diet. These results indicated that ruminal fermentation conditions might be improved and the fermentation of dietary carbohydrates was modified by Chinese herbal medicine. Angelica root and Atractylodes rhizome contain considerable amounts of essential oils which have been well recognized as the main bioactive components of the two herbs [24,25]. Both *in vitro* and *in vivo* studies have shown that essential oils could favorably manipulate rumen fermentation by changing VFA production. Castillejos *et al*. [26,27] reported that essential oils could increase total VFA production and acetate proportion in continuous culture fermenters. The feeding effects of essential oils in growing lambs were studied by Chaves *et al*. [28], who observed the significantly higher concentration of rumen total VFA in the experimental groups than in the control group. Similar results can also be found in dairy cows [29]. These research findings may clarify the observation of our current experiment, where the changes in rumen VFA concentration might be largely due to the effect of essential oils in CHM-diet.

The NH_3_ is produced as the product of dietary nitrogenous substances through microbial fermentation in the rumen. In the present study, rumen NH_3_ concentration remained similar between diets. Castillejos *et al.*[30] reported that essential oils had no influence on NH_3_ concentration of rumen fluid in a long term *in vitro* incubations. Similar response on rumen NH_3_ concentration was also observed in cows by Yang *et al.*[31]. In addition, various reports have verified that rumen NH_3_ concentration was influenced by dietary CP intake [32,33]. Because the addition of Chinese herbal medicine was small (only 2%) in our current experiment, the lack of change in rumen NH_3_ concentration might also be partly related to a comparable CP intake between dietary treatments.

Rumen pH values were within the normal range for both dietary treatments. The lower rumen pH for CHM-diet might be associated with the higher rumen VFA concentration, which agrees with Salman *et al*. [34] who demonstrated that rumen pH values were contrary to total VFA concentration in goats.

### Plasma NEFA and free amino acids

Plasma NEFA is an important indicator of nutritional status in farm animals, which can directly indicate the adverse responses to such as fasting, negative energy balance or stress [35-37]. In our present study, concentration of plasma NEFA did not differ between diets and the values were presented within normal range for sheep. It indicated that the sheep fed on CHM-diet had comparable nutritional status with those fed on MH-diet without causing any adverse response or stress.

El-Shafei *et al*. [38] supplemented Astragalus root to broiler chick diets, and reported that the total protein in serum significantly increased probably due to a hormonal regulating effect of Astragalus root on protein metabolism. In association with the current experiment, although the level of total protein in blood was not determined, the trend of higher concentrations of certain plasma amino acids and total amino acid might be related to the bioactivity of herbs in CHM-diet.

### Plasma glucose and protein kinetics

Based on the knowledge from human research, it has been well-known that the Chinese herbal medicine mixture is able to induce hematopoiesis by accelerating the generation, growth and maturity of blood cells [39]. Although it was expected that plasma glucose metabolism would be enhanced by the Chinese herbal medicine in sheep due to its bioactive properties on hematopoiesis, no positive impact was found on either plasma glucose concentration or plasma GluTR in the current experiment. In ruminants, because the dietary carbohydrates are fermented to VFA by microbes in the rumen, most of the glucose must be supplied through gluconeogenesis. Therefore, the supply of glucose precursor is considered as the major factor to influence glucose metabolism [40]. In our present study, propionate, the major glucogenic substrate produced in the rumen, did not differ between diets. It indicated that glucose precursors from the rumen were similar and thus the gluconeogenesis might be comparable between dietary treatments. Al-Mamun *et al*. [41] reported that plasma glucose concentration and turnover rate did not differ in sheep fed on plantain herb (a perennial herb having anti-bacterial and anti-inflammatory properties) or mixed hay, which is in accordance with our present findings.

Plasma LeuTR and the WBPS did not differ between diets. Nevertheless, it was found that higher numerical values of plasma LeuTR resulted in lower numerical values of the WBPS for CHM-diet than for MH-diet. This may be largely due to the calculation method as we used plasma LeuTR and LeuOX to calculate the WBPS, where the differences in LeuOX were greater than those in LeuTR. Similar data can also be found in a previous report of Sano *et al*. [42]. So far, little information is available regarding the performance of Chinese herbal medicine on plasma amino acid kinetics and protein synthesis in ruminants. The data obtained from human research is also limited. Al-Mamun *et al*. [43] reported that plasma LeuTR decreased but the WBPS remained unchanged in sheep fed on plantain herb compared with fed on mixed hay, which partly agrees with our present findings. Li *et al.*[44] used [^15^ N]glycine as the tracer to determine the effect of a Chinese herbal medicine mixture (Astragalus root and Angelica root, same as the present study) on protein metabolism in nephrotic patients, and observed that Chinese herbal medicine could improve the disorder of protein metabolism and increase the level of serum protein by improving the net rate of protein synthesis. However, their findings are inconsistent with the results of our current experiment in sheep.

Taken together, the sheep fed on CHM-diet did not enhance plasma GluTR, LeuTR and WBPS as expected under the conditions of the present study. In the treatment of human diseases, Chinese herbal medicine is normally taken as syrup of its extract. The herbs are processed by boiling in order to remove foreign substances and reduce toxic contents as well as increase therapeutic effects [39]. During the feeding periods of our current experiment, the Chinese herbal medicine was directly given to the animals without any treatment, thus the lack of changes in GluTR, LeuTR and WBPS between dietary treatments might be partly related to the factor that we did not process the Chinese herbal medicine, so that it was not as effective as expected.

### Heat production

Until now, to our knowledge, the effect of Chinese herbal medicine on heat production has not been investigated either in humans or in animals. Heat is defined as the released energy that is produced from the oxidation process of the nutritive substances such as carbohydrate, protein and fat in the body. In our present study, metabolic heat production tended to be greater for CHM-diet than for MH-diet. The trend of increased heat production indicated that Chinese herbal medicine might play a role in accelerating nutrients oxidation in sheep. It is in good accordance with the result of LeuOX, which also tended to be greater for CHM-diet than for MH-diet. Furthermore, the sheep fed on CHM-diet had a higher body weight gain than those fed on MH-diet. Because energy is essential to animals in almost all life activities including maintenance, growth and production, the trend of increased heat production also demonstrated that the CHM-diet could provide more available energy for growth than MH-diet in sheep.

## Conclusions

Although no significant effect on plasma glucose and protein metabolism was found, the sheep fed on CHM-diet had a higher body weight gain and showed positive impacts on rumen fermentation and energy metabolism without resulting in any adverse response than those fed on MH-diet. Therefore, these results suggested that the Chinese herbal medicine mixture should be considered as a potential feed additive for sheep.

## Competing interests

The authors declare that they have no competing interests.

## Authors’ contributions

XL, as the lead author, was in charge of all research work, including designing the protocol, carrying out the experiment and writing the manuscript. KY, MK, XB and AP participated in the lab work to perform the chemical analysis of all samples, and provided kind suggestions for the manuscript. HS, as the supervisor to the lead author, was involved in the design and execution of the study, and approved the final manuscript. All authors read and approved the final manuscript.
